# Improved trends of lung cancer mortality‐to‐incidence ratios in countries with high healthcare expenditure

**DOI:** 10.1111/1759-7714.13912

**Published:** 2021-04-07

**Authors:** Wen‐Wei Sung, Kwong‐Kwok Au, Han‐Ru Wu, Chia‐Ying Yu, Yao‐Chen Wang

**Affiliations:** ^1^ Department of Urology Chung Shan Medical University Hospital Taichung Taiwan; ^2^ School of Medicine, Chung Shan Medical University Taichung Taiwan; ^3^ Institute of Medicine, Chung Shan Medical University Taichung Taiwan; ^4^ Division of Thoracic Surgery, Department of Surgery Chung Shan Medical University Hospital Taichung Taiwan; ^5^ Division of Pulmonary Medicine, Department of Internal Medicine Chung Shan Medical University Hospital Taichung Taiwan

**Keywords:** expenditure, incidence, lung cancer, mortality, mortality‐to‐incidence ratio

## Abstract

**Background:**

Lung cancer stage has a significant impact on prognosis, and early detection of lung cancer relies on screenings. Despite the strong relationship between screening and lung cancer staging, the role of healthcare expenditure in lung cancer outcomes remains unknown. The aim of this study was to evaluate the relationship between economic status and clinical outcomes in lung cancer.

**Methods:**

Data were obtained from GLOBOCAN and the World Health Organization. Mortality‐to‐incidence ratios (MIRs) and their change over time, calculated as the difference between the MIRs of 2012 and 2018 (*δ*MIR), were used to evaluate their correlation to expenditures on healthcare and human development index (HDI) disparities via Spearman's rank correlation coefficient.

**Results:**

Regions such as North America have relatively high crude incidence rates but low MIR values. Furthermore, countries with lower crude incidence rates spent less on healthcare. The results show significant negative associations between HDI, current health expenditure (CHE) per capita, CHE as a percentage of gross domestic product (CHE/GDP), and MIR. As for MIR and *δ*MIR, countries with favorable MIRs also showed improving MIRs based on *δ*MIR.

**Conclusions:**

HDI, CHE per capita, CHE/GDP, and development status play noticeable roles in the prognosis of lung cancer, leading to large disparities in clinical outcomes.

## Graphical abstract

In the present study, we investigated the association between mortality‐to‐incidence ratios (MIRs) and healthcare expenditure in lung cancer in 60 countries. Countries with the lowest crude incidence rates were found to spend less on healthcare. The results also showed significant negative associations between the human development index (HDI), current health expenditure (CHE) per capita, CHE as a percentage of gross domestic product (CHE/GDP) and MIR. As for MIR and δMIR, countries with favorable MIRs also had improved MIRs based on δMIR. HDI, CHE per capita, CHE/GDP and development status all play a noticeable part in the prognosis of lung cancer, leading to a large disparity in clinical outcomes.

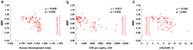

## INTRODUCTION

Lung cancer is the most commonly diagnosed cancer worldwide. Worldwide, 2 093 876 new cases of lung cancer were reported in 2018, accounting for 11.6% of total cancer diagnoses. Furthermore, lung cancer is the leading cause of cancer deaths.^1^ A total of 1 761 007 deaths from lung cancer were reported in 2018, accounting for 18.4% of total cancer deaths.^1^ Median survival after diagnosis has been reported to be 13 months for nonmetastatic lung cancer and only five months for its metastatic variants.^2^ Although many advanced therapies have been developed in recent years, including those using new surgical techniques, chemotherapy, radiotherapy, targeted interventions, and immunotherapy, lung cancer still presents a poor prognosis. Together, these conditions highlight the importance of lung cancer research.

According to previous epidemiological studies, regional differences related to development status exist in lung cancer‐related data. The incidence of lung cancer is higher in developed and fast‐growing economies.^3^ Comprehension of the association between healthcare expenditure and MIR can provide health program executors useful information to plan medical investments in lung cancer, especially in countries with a higher incidence of lung cancer.

Additionally, the prognosis for lung cancer patients is dramatically associated with the stage at diagnosis. The five‐year survival rates are 57% in the localized stage, 31% in the regional stage, and 5% in the distant stage.^4^ Unfortunately, few people are diagnosed at the regional stage or earlier. These data demonstrate the importance of early diagnosis as a good strategy for reducing lung cancer mortality; early screening is therefore of great importance. A previous study showed that the use of low dose computed tomography (LDCT) reduced lung cancer mortality by 20%.^5^ The mortality‐to‐incidence ratio (MIR) can therefore act as an effective evaluation tool for early screenings. Furthermore, the widespread use of early screening has much to do with the local investment in medical devices. Our study was conducted to clarify the association between the human development index (HDI) ranking, current health expenditure (CHE) per capita, CHE as a percentage of gross domestic product (CHE/GDP), and the crude rates of incidence and mortality for lung cancer in order to gain a better understanding of whether a country with more medical resources will have a lower MIR and change the trend of MIR over time.

## METHODS

Epidemiological lung cancer (ICD‐10 codes C33–34) data were obtained from the GLOBOCAN database, a public access database that provides cancer epidemiology estimates from 2012 and 2018 for 185 countries (https://gco.iarc.fr/today/). The data on health expenditures, including per capita CHE and CHE/GDP, were obtained from the World Health Statistics database (https://www.who.int/gho/publications/world_health_statistics/en/). The HDIs were obtained from the United Nations Development Programme, Human Development Report Office (http://hdr.undp.org/en).

MIR was defined as the ratio of the crude rate of mortality to the crude rate of incidence, as previously described.^6^, ^7^, ^8^, ^9^ *δ*MIR was defined as the difference between the MIRs of 2012 and 2018 (*δ*MIR = MIR [in 2012] – MIR [in 2018]).^10^ The exclusion criteria for country selection included data missing in the World Health Organization statistics (*N* = 12), missing HDI data (*N* = 2), a data quality report in GLOBOCAN 2012 (*N* = 110,^11^ and outliers for the MIR (*N* = 1). A total of 60 countries were included in the final analysis.

The associations between the MIR, *δ*MIR, and other factors among the various countries were estimated using Spearman's rank correlation coefficients, which were calculated with SPSS statistical software (version 15.0, SPSS, Inc.). Values of *p* < 0.05 were considered statistically significant. Scatterplots were generated using SigmaPlot.

## RESULTS

### Numbers, CRs, ASRs, and MIRs of new cases and deaths from lung cancer according to continents

The numbers, crude rates (CRs), and age standardized rates (ASRs) of new cases of lung cancer by continent are summarized in Table 1. The highest number of new cases occurred in Asia, and the lowest in Oceania (1 146 776 and 14 799, respectively). In terms of the CRs of new cases, North America presented the highest CR, while Africa showed the lowest cumulative risk (64.0 and 3.0, respectively). Interestingly, the ASR of new cases decreased dramatically in America compared to the CR. The number, CR, and ASR of deaths also followed the aforementioned pattern. As for the MIR, the highest occurred in Africa and the lowest was noted in North America (0.93 and 0.65, respectively).

**TABLE 1:** Summary of the number, crude rank (CR), age standardized rate (ASR) and mortality‐to‐incidence ratio (MIR) of lip and oral cancer by region

	New cases			Deaths			MIR
Region	Number	CR	ASR	Number	CR	ASR	
Africa	13 324	1.0	1.7	9066	0.7	1.2	0.71
Asia	220 810	4.9	4.2	124 900	2.8	2.4	0.57
Europe	57 737	8.0	4.3	21 834	3.0	1.6	0.38
Latin America and the Caribbean	18 525	2.9	2.6	7050	1.1	1.0	0.38
North America	25 354	7.1	4.2	4424	1.2	0.7	0.17
Oceania	4163	10.2	7.5	895	2.2	1.5	0.22

### HDI, CHE, and cancer incidence and mortality in selected countries

Table 2 summarizes the HDIs, CHEs, and CRs of incidence and mortality in selected countries. Regarding crude incidence, the CR exceeded 75 in Serbia, Denmark, Belgium, and Germany (91.1, 80.8, 77.5, and 76.9, respectively). Apart from Serbia, these countries also had relatively high HDIs (0.799, 0.930, 0.919, and 0.939, respectively). In addition, all these countries also exhibited a CHE/GDP ratio greater than 9%. Conversely, the country with the lowest crude incidence rate was Oman, which spent less on healthcare (CHE = 3.8% of GDP).

**TABLE 2:** Summary of the human development index (HDI), current health expenditure (CHE), cancer incidence, cancer mortality, and mortality‐to‐incidence ratio (MIR) in lip and oral cancer (*N* = 61)

	HDI	CHE		Incidence			Mortality			
Country		Per capita	% of GDP	Number	CR	ASR	Number	CR	ASR	MIR
Argentina	0.830	998	6.8	1357	3.1	2.5	512	1.2	0.9	0.39
Australia	0.938	4934	9.4	2682	11.1	6.9	378	1.6	0.9	0.14
Austria	0.914	4536	10.3	488	5.7	3.0	232	2.7	1.4	0.47
Bahrain	0.838	1190	5.2	16	1.0	1.5	10	0.6	1.0	0.64
Belarus	0.817	352	6.1	653	7.0	4.1	316	3.4	2.0	0.49
Belgium	0.919	4228	10.5	923	8.3	4.7	305	2.7	1.4	0.33
Brazil	0.761	780	8.9	9902	4.7	4.0	3965	1.9	1.6	0.40
Bulgaria	0.816	572	8.2	459	6.7	3.4	164	2.4	1.2	0.36
Canada	0.922	4508	10.4	2633	7.3	3.9	594	1.6	0.8	0.22
Chile	0.847	1102	8.1	275	1.5	1.1	113	0.6	0.4	0.42
Colombia	0.761	374	6.2	775	1.6	1.4	308	0.6	0.5	0.39
Costa Rica	0.794	929	8.1	78	1.6	1.2	35	0.7	0.5	0.44
Croatia	0.837	852	7.4	291	7.2	3.8	117	2.9	1.5	0.40
Cuba	0.778	826	10.9	1238	11.0	6.2	380	3.4	1.8	0.31
Cyprus	0.873	1563	6.8	29	2.5	1.6	8	0.7	0.5	0.27
Czechia	0.891	1284	7.3	859	8.3	4.3	361	3.5	1.8	0.42
Denmark	0.930	5497	10.3	397	7.1	3.8	125	2.2	1.1	0.31
Ecuador	0.758	530	8.5	217	1.3	1.2	82	0.5	0.5	0.38
Egypt	0.700	157	4.2	1295	1.3	1.6	352	0.4	0.4	0.28
Estonia	0.882	1112	6.5	79	6.2	3.3	40	3.1	1.7	0.50
Fiji	0.724	175	3.6	27	3.0	2.9	11	1.2	1.2	0.40
Finland	0.925	4005	9.4	348	6.5	3.0	116	2.2	0.9	0.34
France	0.891	4026	11.1	6815	10.8	6.2	1516	2.4	1.3	0.22
Germany	0.939	4592	11.2	7271	9.1	4.4	2311	2.9	1.3	0.32
Iceland	0.938	4375	8.6	13	3.9	2.3	3	0.9	0.5	0.23
Ireland	0.942	4757	7.8	275	5.8	3.7	74	1.6	0.9	0.28
Israel	0.906	2756	7.4	155	1.9	1.4	51	0.6	0.4	0.32
Italy	0.883	2700	9.0	3500	6.1	2.7	1184	2.1	0.9	0.34
Jamaica	0.726	294	5.9	52	1.8	1.5	15	0.5	0.4	0.29
Japan	0.915	3733	10.9	8138	6.7	2.8	2496	2.1	0.7	0.31
Kuwait	0.808	1169	4.0	59	1.4	2.0	33	0.8	1.1	0.56
Latvia	0.854	784	5.8	239	12.7	6.8	113	6.0	3.2	0.47
Lithuania	0.869	923	6.5	188	6.7	3.7	114	4.1	2.3	0.61
Luxembourg	0.909	6236	6.0	45	7.8	4.8	10	1.7	1.0	0.22
Malaysia	0.804	386	4.0	640	2.0	2.0	302	1.0	1.0	0.48
Malta	0.885	2304	9.6	18	4.2	1.9	6	1.4	0.4	0.33
Mauritius	0.796	506	5.5	62	4.9	3.3	28	2.2	1.5	0.45
Netherlands	0.934	4746	10.7	1418	8.5	4.4	257	1.5	0.7	0.18
New Zealand	0.921	3554	9.3	264	5.7	3.4	58	1.2	0.7	0.21
Norway	0.954	7464	10.0	362	6.9	3.7	68	1.3	0.7	0.19
Oman	0.834	636	3.8	65	1.3	2.0	31	0.6	1.1	0.49
Philippines	0.712	127	4.4	1372	1.3	1.6	673	0.6	0.8	0.48
Poland	0.872	797	6.3	3203	8.6	4.8	1505	4.0	2.2	0.47
Portugal	0.850	1722	9.0	817	8.2	4.3	269	2.7	1.4	0.33
Qatar	0.848	2030	3.1	26	1.0	2.0	14	0.5	1.4	0.54
Russian Federation	0.824	524	5.6	9340	6.6	4.0	5443	3.8	2.3	0.58
Serbia	0.799	491	9.4	701	8.1	4.9	261	3.0	1.7	0.37
Singapore	0.935	2280	4.3	199	3.5	1.9	85	1.5	0.8	0.43
Slovakia	0.857	1108	6.9	541	10.1	6.0	224	4.2	2.5	0.42
Slovenia	0.902	1772	8.5	135	6.7	3.5	47	2.3	1.1	0.34
South Africa	0.705	471	8.2	1328	2.3	2.7	829	1.4	1.7	0.61
South Korea	0.906	2013	7.4	1467	2.9	1.6	507	1.0	0.5	0.34
Spain	0.893	2354	9.2	3843	8.6	4.0	969	2.2	1.0	0.26
Sweden	0.937	5600	11.0	556	5.7	2.8	145	1.5	0.7	0.26
Switzerland	0.946	9818	12.1	639	7.7	4.0	184	2.2	1.1	0.29
Thailand	0.765	217	3.8	4169	6.1	3.8	2159	3.2	2.0	0.52
Trinidad and Tobago	0.799	1146	6.0	42	3.1	2.2	20	1.5	1.1	0.48
Ukraine	0.750	125	6.1	3358	7.8	4.4	2058	4.7	2.8	0.60
United Kingdom	0.920	4356	9.9	5645	8.7	4.9	1443	2.2	1.1	0.25
United States of America	0.920	9536	16.8	22 715	7.1	4.2	3830	1.2	0.7	0.17
Uruguay	0.808	1281	9.2	149	4.4	2.9	70	2.1	1.3	0.48

Abbreviations: ASR, age standardized rate; CR, crude rate; GDP, gross domestic product.

### MIRs in 2018 and MIR disparities between 2012 and 2018

The MIRs in 2018 and MIR disparities between 2012 and 2018 are summarized in Table 2. Three countries exhibited a MIR no greater than 0.65 for 2018, namely Australia, the United States, and Japan (0.65, 0.64, and 0.62, respectively). Based on *δ*MIR, they also showed improved MIRs. The highest MIR in 2018 was presented by Oman (0.96), which also had the lowest crude incidence rate as well as a negative *δ*MIR.

### Significant association of HDI and CHE with the crude rates of incidence/mortality, MIR, and δMIR

**FIGURE 1:**
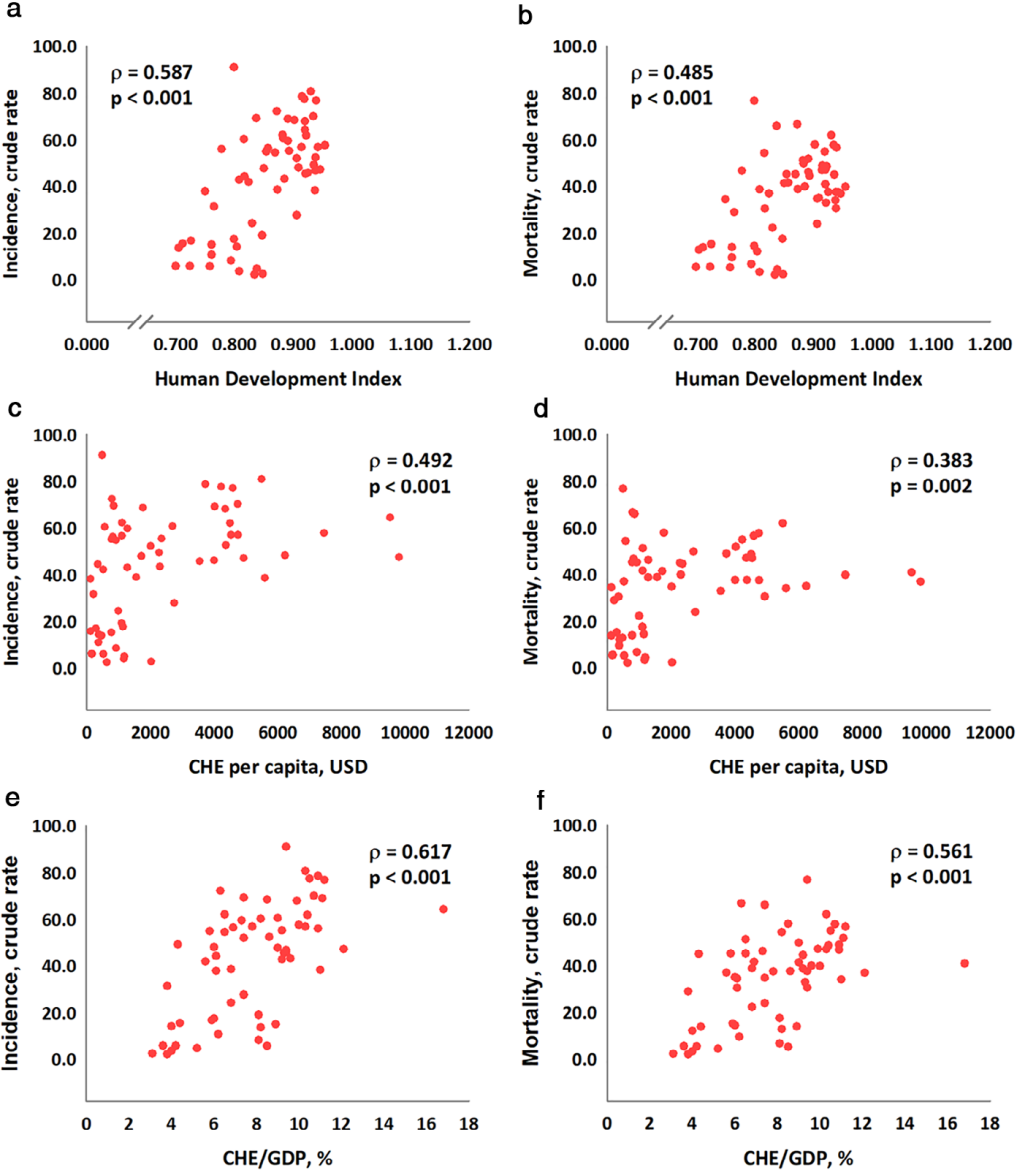
Association between human development index, current health expenditure and crude rate of incidence (a, c, and e, respectively) and mortality (b, d, and f, respectively) in lung cancer

**FIGURE 2:**
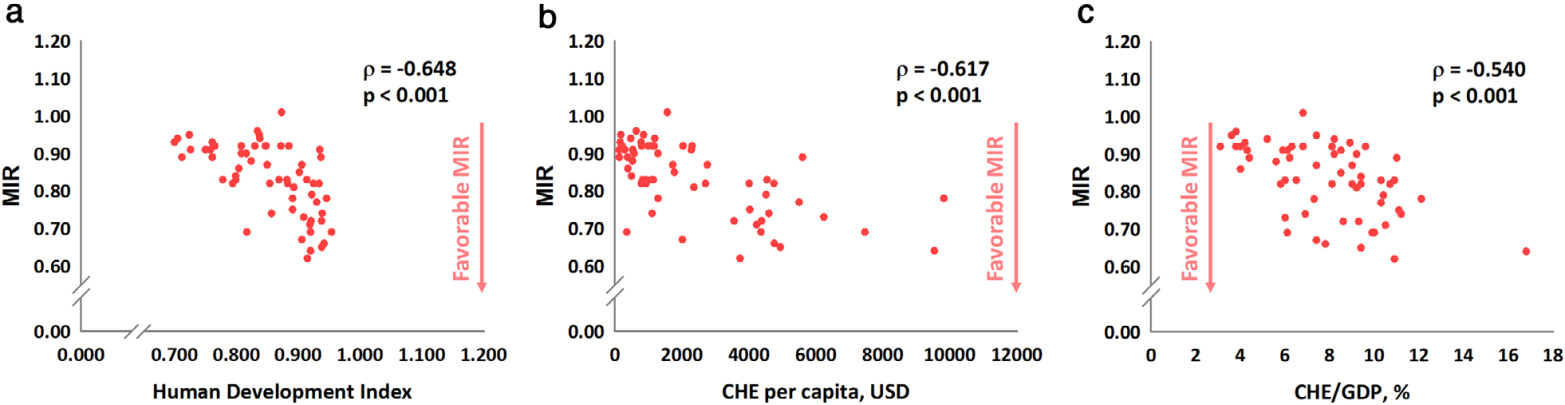
Significant associations of (a) human development index, (b) current health expenditure per capita, and (c) current health expenditure as a percentage of gross domestic product with mortality‐to‐incidence ratio in lung cancer

**FIGURE 3:**
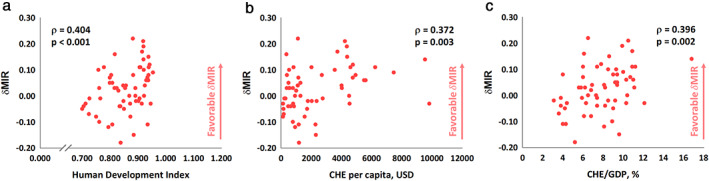
Significant associations of (a) human development index, (b) current health expenditure per capita, and (c) current health expenditure as a percentage of gross domestic product with the difference between the mortality‐to‐incidence ratios of 2012 and 2018 for lung cancer

Figure 1 shows the linear correlations among HDI, CHE per capita, CHE/GDP, and crude incidence and mortality rates. HDI, CHE per capita, and CHE/GDP showed significant positive associations with the crude incidence rate (ρ = 0.587, *p* < 0.001, Figure 1(a); ρ = 0.492, *p* < 0.001, Figure 1(c); and ρ = 0.617, *p* < 0.001, Figure 1(e), respectively). The same situation was noted for the mortality rate (ρ = 0.485, *p* < 0.001, Figure 1(b); ρ = 0.383, *p* = 0.002, Figure 1(d); and ρ = 0.561, *p* < 0.001, Figure 1(f), respectively). Conversely, HDI, CHE per capita, and CHE/GDP showed significant negative associations with MIR (ρ = −0.648, *p* < 0.001, Figure 2(a); ρ = −0.617, *p* < 0.001, Figure 2(b); and ρ = −0.540, *p* < 0.001, Figure 2(c), respectively). Finally, HDI, CHE per capita, and CHE/GDP showed significant associations with the improvement of MIR between 2012 and 2018, named δMIR.(ρ = 0.404, p < 0.001, Figure 3(a); ρ = 0.372, p = 0.003, Figure 3(b); and ρ = 0.396, p = 0.002, Figure 3(c), respectively).

## DISCUSSION

Our study illustrates the relationship of MIR with HDI, CHE per capita, and CHE/GDP. The results indicate that the amount of available medical resources affects lung cancer prognosis. The greater the expenditure on medical services, the better the medical treatments that can be provided. Developed countries are able to spend more money on screening tools such as computed tomography (CT), perform more kinds of advanced surgeries involving, for example, video‐assisted techniques, and provide more precise therapies such as image‐guided radiotherapy (IGRT) and immunotherapy.^7^, ^12^, ^13^ All these therapies show benefits on survival.^7^

The incidence and mortality rates for lung cancer are relatively high in developed countries such as North America. According to one study, lung cancer transformed from a rare disease to a dominant form of cancer at the start of the 20th century. Therefore, the evidence points to the possibility of greater economic development leading to an increased risk for lung cancer.^14^ Interestingly, the age‐standardized rates of new cases and deaths dropped dramatically in North America as compared to the crude rate. A previous study reported that the risk of 70 year‐old men developing lung cancer in the next 10 years is 35 times higher than that for 40 year‐old men.^15^ This finding reflects that aging is a contributory factor in high incidence and death crude rates (Figure 3).

In countries with high economic status, healthcare policies pertaining to lung cancer should be considered carefully due to the higher incidence and mortality for lung cancer. The results of this study are in agreement with those of previous studies reporting that HDI, CHE per capita, and CHE/GDP show significantly negative associations with MIRs. As expected, the more developed countries showed lower MIRs, the lowest occurring in North America, and the highest, in Africa. Countries with more medical funds do show better clinical outcomes for lung cancer. CT screening may play a role in these results. Many studies show that CT screening is very helpful for lung cancer prognoses. For example, 50% of all early‐stage lung cancers show small, subcentimeter lung nodules in CT scans, facilitating the diagnosis of lung cancer at an early stage.^14^ Countries with a favorable economic status may be better placed to popularize CT usage. Additionally, countries with higher HDI not only showed favorable MIRs, but also presented improved MIR trends over time. This result lends additional confidence to our belief that lung cancer prognosis follows economic status and medical investment trends.

In conclusion, sufficient medical investments can help decrease the gap between the current medical situation and future objectives, which can also explain the positive correlations between MIR disparities and HDI, CHE per capita, and CHE/GDP. Less developed regions showed no significant improvement in lung cancer prognosis. This result might be attributed to the insufficient medical resources in those countries.

To the best of our knowledge, our study is the first to highlight the association among the lung cancer MIR, HDI, CHE per capita, and CHE/GDP. However, our research also has some limitations. First, we excluded some countries with insufficient data, especially those with a low HDI ranking. This may have slightly impacted the comprehensiveness of the study. Second, detailed categories of lung cancer types and risk factors, such as smoking history, were not available in our study. Third, medical investments in lung cancer care cannot be entirely represented by HDI, CHE per capita, and CHE/GDP. Fourth, another study found that MIR can be mistaken as an indication of survival. However, while MIRs may not be able to completely replace a survival cohort, they are still worth analyzing, considering that global data on survival cohorts are unavailable.^16^ Despite these limitations, our study demonstrates that lung cancer has higher incidence and mortality values as well as lower MIRs in more developed regions.

## CONFLICT OF INTEREST

The authors declare that they have no competing interests.
